# Safety Profile, Toxicokinetic, and Intestinal Absorption Differences of a Naturally-Derived Anti-Rheumatic Drug, Sinomenine Hydrochloride, in Normal and Arthritic Rats

**DOI:** 10.3390/pharmaceutics17040484

**Published:** 2025-04-07

**Authors:** Yini He, Hong Huang, Gejing Li, Ye Zhang, Junjie He, Ye Lin, Feichi Wu, Jianye Yan, Xiong Cai, Liang Liu

**Affiliations:** 1 Institute of Innovation and Applied Research in Chinese Medicine, School of Chinese Medical Sciences, Hunan University of Chinese Medicine, Changsha 410208, China; 20223595@stu.hnucm.edu.cn (Y.H.); 20203727@stu.hnucm.edu.cn (H.H.); 20223585@stu.hnucm.edu.cn (G.L.); zy510@stu.hnucm.edu.cn (Y.Z.); jjhe@stu.hnucm.edu.cn (J.H.); linye@hnucm.edu.cn (Y.L.); yanjy@hnucm.edu.cn (J.Y.); 2 State Key Laboratory of Traditional Chinese Medicine Syndrome, Guangdong Provincial Hospital of Chinese Medicine, Guangdong Provincial Academy of Chinese Medical Sciences, The Second Affiliated Hospital of Guangzhou University of Chinese Medicine, Guangzhou 510006, China; 3 Hunan ZhenQin Pharmaceutical Group Co., Ltd., Huaihua 418000, China; zq_jszx@163.com

**Keywords:** sinomenine hydrochloride, adjuvant-induced arthritis, safety profile, toxicokinetic, intestinal absorption, P–glycoprotein

## Abstract

**Background/Objective**: Sinomenine hydrochloride (SH), a natural anti–rheumatic drug derived from the Chinese medicinal plant *Sinomenium acutum*, demonstrates disease–modifying properties but lacks comprehensive safety and toxicokinetic (TK) comparisons between physiological and pathological states. This study evaluated SH’s safety profile, TK parameters, and intestinal absorption differences in adjuvant–induced arthritis (AIA) and normal rats. **Methods**: Safety assessments determined median lethal doses (LD_50_) in female Sprague Dawley rats. TK parameters were analyzed via a validated ultrahigh performance liquid chromatography-tandem mass spectrometry approach after single oral administration of 600 mg/kg SH. Plasma protein binding (PPB) were measured using equilibrium dialysis. Intestinal absorption was evaluated through everted gut sac experiments, with P–glycoprotein (P–gp) inhibition tested via verapamil co–administration. **Results**: LD_50_ values revealed AIA rats tolerated SH better than normal rats (1179 vs. 805 mg/kg). TK analysis showed that C_max_, AUC_(0-t)_, and AUC_(0-∞)_ of SIN in normal rats were 2.01, 1.94, and 2.14 times higher than in AIA rats, respectively, while CL/F and V/F in AIA rats were 2.24 times greater. In addition, the PPB of SIN in normal rats was 2 times greater than that in AIA rats. AIA rats exhibited significantly lower SH absorption in the jejunum and ileum compared to normal rats. Notably, verapamil co–administration markedly increased SH absorption across most intestinal segments. **Conclusions**: Pathological states significantly alter SH’s safety and TK profiles. Enhanced tolerance in AIA rats correlates with reduced intestinal absorption via altered P–gp activity and decreased PPB. These findings emphasize the necessity of disease–specific evaluations for optimizing SH’s therapeutic safety in pathological contexts.

## 1. Introduction

Sinomenine (SIN) is a bioactive alkaloid monomer extracted from the Chinese medicinal plant *Sinomenium acutum*, with its medicinal form primarily used as hydrochloride [1]. SIN has the molecular formula C_19_H_23_NO_4_ and a molecular weight of 329.39 [2]. A wide range of pharmacological properties has been reported for SIN, including antiarrhythmic [3], antitumor [4], anti–inflammatory [5,6], immunosuppressive [7], and neuroprotective activities [8,9]. As a natural disease-modifying antirheumatic drug (nDMARD), sinomenine hydrochloride (SH), marketed as Zhengqing Fengtongning (ZQFTN), is widely used for the treatment of rheumatoid arthritis (RA) [10].

Pharmacokinetic (PK) variability is a well–recognized factor influencing drug responses and toxicity across populations. Recent studies suggest that PK variability can result from diverse factors. Clinical research has shown that inflammation can significantly alter the PKs of drugs in adults [11,12,13]. Specifically, inflammation–induced upregulation or downregulation of membrane transport proteins can influence the distribution volume of drugs [14,15]. Furthermore, inflammation is a critical regulator of drug metabolism enzymes and transporters, contributing to their variability [16,17]. Drug disposition is also dependent on P–glycoprotein (P–gp), an energy–dependent transporter that facilitates drug excretion into bile and accelerates hepatobiliary elimination. P-gp is expressed in tissues such as the gastrointestinal tract, where it limits oral drug absorption [18]. Notably, P–gp expression levels differ by sex, with significantly lower expression in male rats compared to females [19]. The binding rate of SIN in rat and rabbit plasma or albumin solutions exceeds 60%, while the rate in 1–acid–glycoprotein solutions ranges from 20% to 33% [20]. Moreover, the levels of plasma protein binding (PPB) were altered by inflammation [21,22], fluid transfer and reduced intestinal acidity [23].

During the analysis of the acute toxicity effects of SH, it was observed that normal rats were remarkably more sensitive to SH compared to those with adjuvant–induced arthritis (AIA). This finding suggests that the tolerance to SH differs between physiological and pathological states. In toxicology, a slower metabolic rate and higher blood concentrations of parent compounds are known to contribute to increased toxicity [24]. To address this phenomenon, the present study probed the toxicokinetic (TK) characteristics of SH following oral administration and explored the underlying mechanisms driving these differences, focusing on intestinal absorption and PPB of SH. A robust and validated ultra-high performance liquid chromatography–tandem mass spectrometry (UPLC–MS/MS) approach was employed to evaluate the TK properties, intestinal absorption, and in vitro PPB of SH.

## 2. Materials and Methods

### 2.1. Chemicals

Mineral oil (MKCN4267) was procured at Sigma–Aldrich (Shanghai, China). *Mycobacterium tuberculosis* H37Ra (3089150) was obtained from Becton Dickinson (Franklin Lakes, NJ, USA). Sinomenine (purity ≥ 98%, YR–Q0013220619) was purchased at the Baoji Herbest Bio–Tech Co., Ltd. (Baoji, Shanxi, China), while SH (purity ≥ 98%, YK–220508) was provided by the Hunan ZhenQin Pharmaceutical Group (Huaihua, Hunan, China). Pentoxifylline (purity ≥ 98%, 100,591–202203), served as the internal standard (IS), was acquired from the National Institute of Control of Pharmaceutical and Biological Products (Beijing, China). Verapamil hydrochloride (M08IS214501) was sourced from Yuanye Biotechnology Co., Ltd. (Shanghai, China). Sodium chloride solution (0.9%, D23081202) was provided by Hunan Kelun Pharmaceutical Co., Ltd. (Yueyang, Hunan, China). LC–MS–grade formic acid (214911) and methanol (222635), as well as Rapid Equilibrium Dialysis (RED) single–use plates with inserts (YG9001543), were purchased from Thermo Fisher Scientific (Waltham, MA, USA). The remaining chemicals employed in the experiments met analytical grade standards.

### 2.2. Animals

Specific–pathogen–free Sprague Dawley (SD) female rats weighing 70–90 g were procured at the Hunan Slac Jingda Laboratory Animal Co. (Changsha, Hunan, China). Animals were housed in the Laboratory Animal Center of Hunan University of Chinese Medicine (HNUCM). Before the experiments, all rats were acclimated to laboratory conditions for five days. The animals were grouped and maintained under controlled environmental conditions, including a 12 h light/dark cycle, a temperature range of 20–26 °C, and relative humidity of 40–70%. Throughout the study, the rats had unrestricted access to food and water.

### 2.3. Induction and Evaluation of AIA

Arthritis was induced in rats on day 0 by subcutaneous injection of 0.1 mL of complete Freund’s adjuvant (CFA) containing 350 μg of heat-inactivated *Mycobacterium tuberculosis* H37Ra at the base of the tail. Arthritis scores and hind paw volumes were measured every three days from day 9 through day 21 as previously described [25]. Additionally, body weight was monitored every three days. AIA rats exhibited significantly slower increases in body weight, alongside progressively increased arthritis scores and paw volumes. Significant differences in these parameters between AIA and normal rats confirmed the successful establishment of the AIA model.

### 2.4. Assessment of the Acute Toxicity

To study acute toxicity and to investigate differences between physiological and pathological states, normal SD rats were administered SH via intragastric gavage at doses of 400, 800, 1200, and 1600 mg/kg. AIA rats received SH under the same conditions at doses of 400, 600, 800, 1200, 1600, and 2000 mg/kg. Mortality rates and clinical symptoms were recorded and analyzed. The median lethal dose (LD_50_) of SH was calculated employing SPSS software (SPSS 25.0 for Windows).

### 2.5. TK Studies

TK studies were conducted on normal (*n* = 20) and AIA (*n* = 20) rats following a single oral administration of 600 mg/kg SH. Blood samples (0.2 mL) were harvested from the jugular vein at baseline (0 min) and at predetermined time points post–administration (5, 10, 20, 30, 40, 50, 60, 90, 120, 180, 240, 360, 480, and 720 min). Blood samples were centrifuged at 3540× *g* for 15 min at 4 °C within 2 h of collection. The resulting plasma was carefully separated and transferred to Eppendorf tubes, then preserved at −80 °C for subsequent analysis.

### 2.6. Preparation of Everted Intestinal Sacs

Everted intestinal sacs from rats were prepared as previously described [26]. In brief, 24 rats were evenly randomized into four groups, i.e., normal, normal plus verapamil hydrochloride, AIA, and AIA plus verapamil hydrochloride. The rats underwent a 12 h fasting period followed by intraperitoneal administration of sodium pentobarbital (50 mg/kg) for anesthesia. The duodenum, jejunum, ileum, and colon were carefully isolated and washed with saline at room temperature. The intestines were carefully everted using glass rods and sealed with silk yarn before being filled with Krebs–Ringer buffer (0.6 mL). One group of intestinal sacs was placed in conical flasks containing 50 mL of SH solution (60 mg/mL in Krebs–Ringer buffer), while another group of sacs was placed in flasks containing a mixture of SH (60 mg/mL) and verapamil hydrochloride (0.25 mg/mL) in Krebs–Ringer buffer. The flasks were incubated at 37 °C with a gas mixture (O_2_/CO_2_, 95%:5%). Following 1 h incubation, the sacs were removed, rinsed with saline, and dried. The sacs were then slit open, and the intestinal fluid (contents of the sacs) was collected. The intestinal absorption of SIN was analyzed by a UPLC–MS/MS.

### 2.7. Plasma Protein Binding

The PPB of SIN was evaluated utilizing a single–use RED plate. The donor (sample chamber) compartment was filled with 200 μL of rat plasma, while the buffer chamber was filled with 400 μL of phosphate buffer. The phosphate buffer consisted of potassium dihydrogen phosphate, dipotassium hydrogen phosphate, and sodium chloride (pH = 7.4) [27]. SIN (4 μg/mL) [20] was added to the sample chamber in duplicate. The plate was sealed and placed on an orbital shaker at 49 g for 4 h at 37 °C to reach equilibrium. Samples were then collected from both the sample and buffer compartments and analyzed by a UPLC-MS/MS.

The percent PPB (%*P*) of SIN was calculated using the following formula:

$$\%P=\frac{C_{S}-C_{B}}{C_{S}}\times 100$$

where *C_S_* and *C_B_* represent the concentrations of the SIN in the sample and buffer chambers, respectively.

### 2.8. Sample Processing

In this study, protein precipitation using acetonitrile was employed to process all biological samples, including rat plasma, intestinal fluid, and plasma protein samples, as well as quality control (QC) and calibration standard samples. For stock solution preparation, 50% methanol solution was used to dissolve SIN (24 mg/mL, 1.00 mg/mL) and IS (60 μg/mL, 1.00 mg/mL). The stock solutions were stored at 4 °C until further use, and subsequent dilutions were performed with 50% methanol to achieve the desired concentrations.

For plasma samples (20 μL each), 60 μL of acetonitrile and 20 μL of IS (300 ng/mL) were added. The samples were centrifuged at 12,783× *g* for 10 min at 4 °C. The resulting supernatants were mixed with 500 μL of mobile phase solution.

For intestinal fluid samples (2 μL each), 28 μL of acetonitrile, and 20 μL of IS (30 μg/mL) were added. The centrifugation conditions were identical to those for plasma samples. The supernatants were combined with 550 μL of mobile phase solution and diluted by 100 times.

For plasma protein samples (20 μL each), 60 μL of acetonitrile and 20 μL of IS (100 ng/mL) were added. After centrifugation under the same conditions as above, and the supernatants were mixed with 100 μL of the mobile phase solution.

The above three types of samples were all filtered employing a 0.22 μm Millipore organic-phase filter.

### 2.9. Analytical Method

In this study, the UPLC–MS/MS analytical system comprised an ACQUITY UPLC series liquid chromatography instrument (Waters, Milford, MA, USA) coupled with a QTRAP 5500 mass spectrometer (AB SCIEX, Waltham, MA, USA). The system was operated using ACQUITY Console and Analyst 1.6.2 software.

Chromatographic separation was achieved on a Waters ACQUITY UPLC BEH C18 column (2.1 × 100 mm, 1.7 μm, Waters Corp., Milford, MA, USA), maintained at 30 °C, using a positive electrospray ionization (ESI) source. The mobile phase consisted of 0.1% formic acid in water (A) and methanol (B), with a gradient procedure as follows: 0–1.0 min at 90% A; 1.0–5.0 min at 10% A; and 5–10 min returning to 90% A. The flow rate was maintained at 0.3 mL/min, and the injection volume for each sample was 2 μL.

Detection was conducted using multiple reaction monitoring (MRM) to measure SIN and IS. The transitions monitored were *m*/*z* 330.2 181.0 for SIN and *m*/*z* 279.2 181.2 for IS [28]. Optimized operating conditions were as follows: curtain gas, spray gas, and collision gas at 35 psi (1 psi ≈ 6.895 kPa), 50 psi and medium, respectively; auxiliary heating gas at 50 psi with source temperature 500 °C; ion spray voltage at 5500 V; dwell time of 10 min.

### 2.10. Method Validation

#### 2.10.1. Selectivity

Selectivity was assessed by analyzing blank Krebs–Ringer buffer samples, blank Krebs–Ringer buffer samples spiked with standards and IS, and rat intestinal sac samples incubated at 37 °C in two different solutions for 1 h. This ensured the absence of endogenous interferences in the analysis.

#### 2.10.2. Calibration Curves

Calibration curve samples were prepared by diluting working solutions to different concentrations in the Krebs–Ringer buffer. The calibration curves were constructed through a weighted linear least–squares regression model by plotting the peak-area ratios of analytes to IS (Y) against their nominal concentrations (X). The lower limit of quantification (LLOQ) was evaluated to ensure both accuracy and precision, with deviations not exceeding 20%.

#### 2.10.3. Precision and Accuracy

Three QC samples at varying concentrations were analyzed six times per concentration on the same day to determine intra–day precision (expressed as relative standard deviation, RSD%) and accuracy (calculated as relative error, RE%). This process was repeated over three consecutive days to test inter–day precision and accuracy.

#### 2.10.4. Recovery and Matrix Effect

The extraction recovery of the analytes was examined via comparing the peak areas of extracted samples at three different concentrations with those of blank specimens spiked with the corresponding concentrations. Matrix effects were evaluated by comparing the peak area ratios of blank specimen extracts spiked with analytes to those of analytes dissolved directly in the mobile phase at the same concentrations. Each test was performed in six replicates to ensure reliability.

#### 2.10.5. Stability

The stability of analytes was evaluated using QC samples under the following conditions: storage at ambient temperature for 24 h, storage at −80 °C for 30 days, and repeated freeze–thaw cycles (12 h freezing/12 h thawing) conducted three times. Each condition was tested in six replicates to ensure the reliability of the results.

### 2.11. Statistical Analysis

Data were expressed as the means ± SD or the means ± SEM based on the specified number of independent trials. TK parameters were calculated utilizing non–compartmental analysis in a Phoenix WinNonlin Software 8.1 (Pharsight, Mountain View, CA, USA). Statistical significance of the parameters was assessed utilizing independent samples *t*–test and Mann–Whitney U test. A *p* < 0.05 signified statistically significant.

## 3. Results

### 3.1. Induction of AIA and Assessment of the Acute Toxicity of SH

**Figure 1:**
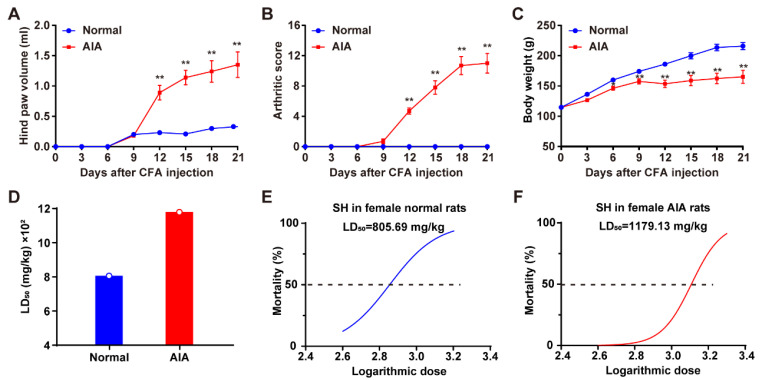
Clinical evaluation of AIA and assessment of the acute toxicity of SH. (**A**) Hind paw volume of rats measured at different times; (**B**) arthritis scores of rats at different times; (**C**) the body weight of normal rats and AIA rats. Data are presented as the means ± SEM (*n* = 6). * *p* < 0.05, and ** *p* < 0.01. (**D**) The median lethal doses (LD_50_) of orally administered SH in SD female normal and AIA rats. (**E**) Logarithmic doses and toxicity curves of SH in normal rats and (**F**) AIA rats.

Following CFA injection, arthritic lesions began to appear in rats between days 9 and 12. Compared with normal rats, significant differences in hind paw volume and arthritic scores became apparent on day 12 (Figure 1A,B). Additionally, AIA rats exhibited significant differences in body weight starting from day 6 post–injection (Figure 1C). The LD_50_ of SH in female normal and AIA rats was determined to be 805.69 mg/kg (95% CI: 554.16–1004.38 mg/kg) and 1179.13 mg/kg (95% CI: 959.19–1472.86 mg/kg), respectively (Figure 1D). Logarithmic doses and toxicity curves of SH are depicted in Figure 1E (normal rats) and 1F (AIA rats). These data indicate significant differences in the tolerance of SD female rats to SH under physiological versus pathological conditions.

### 3.2. Method Validation

#### 3.2.1. Selectivity

**Figure 2:**
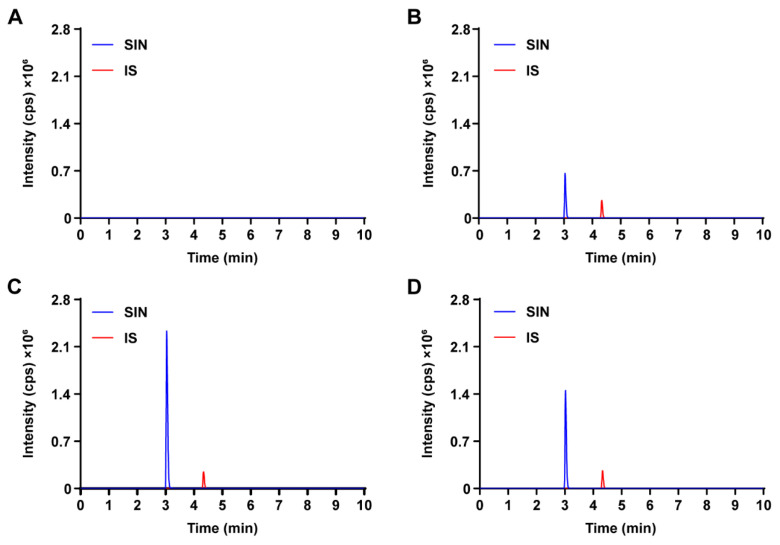
Representative MRM chromatograms of SIN and IS in the intestinal fluid of rats. (**A**) Blank Krebs–Ringer buffer sample; (**B**) blank Krebs–Ringer buffer spiked with SIN and IS; (**C**) intestinal fluid sample were incubated at 37 °C in Krebs–Ringer buffer containing 60 mg/mL SH for 1 h, spiked with IS; (**D**) intestinal fluid sample were incubated at 37 °C in Krebs–Ringer buffer containing 60 mg/mL SH and 0.25 mg/mL verapamil hydrochloride for 1 h, spiked with IS.

Representative chromatograms of blank Krebs–Ringer buffer, blank Krebs–Ringer buffer spiked with SIN and IS, and rat intestinal sac samples incubated at 37 °C in two different solutions for 1 h are summarized in Figure 2. SIN and IS were successfully eluted and separated. The high selectivity of the MRM mode ensured no detectable endogenous interference in SIN analysis.

#### 3.2.2. Linearity and Sensitivity

SIN concentrations in intestines of rats displayed excellent linearity within the quantitation range of 12.3–872 ng/mL. Table 1 presents the linear regression equations and correlation coefficients (r), all of which exceeded 0.99. The LLOQ for all samples was determined to be 12.3 ng/mL, with accuracy and precision meeting predefined acceptance criteria.

#### 3.2.3. Accuracy and Precision

The inter– and intra–day precision and accuracy results are detailed in Table 2. The method demonstrated acceptable performance, with precision ranging from 1.68% to 6.10% and accuracy between 99.50% and 106.03%.

#### 3.2.4. Matrix Effect and Recovery

As shown in Table 2, the extraction recovery rates ranged from 97.03% to 104.05%, while the matrix effects were between 98.15% and 104.26%. These results demonstrated that the method was effective and that there were no significant differences in the matrix effect among the various rat samples.

#### 3.2.5. Stability

Stability data are presented in Table 3. The results show that SIN and IS remained stable under various conditions: at ambient temperature for 24 h (RSD% ranging from 0.87% to 2.25%), after three freeze–thaw cycles (RSD% ranging from 1.23% to 3.17%), and during storage at −80 °C for 30 days (RSD% ranging from 1.67% to 2.07%).

### 3.3. TK Study

**Figure 3:**
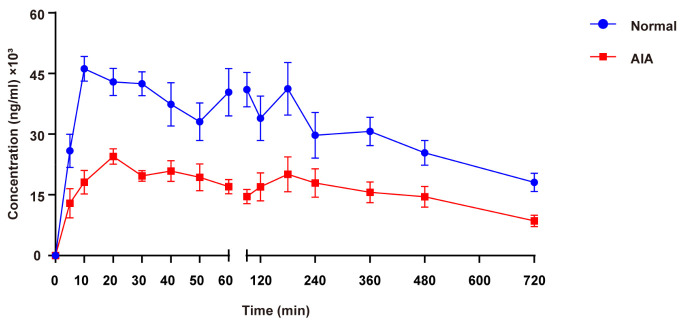
Mean plasma concentration–time curves after oral administration of 600 mg/kg SH in SD female normal and AIA rats. Data are presented as the means ± SEM (*n* = 6).

The mean plasma concentration–time curves for normal and AIA rats following oral administration of SH are illustrated in Figure 3, with the corresponding TK parameters summarized in Table 4. The plasma concentration–time curves indicated that SIN exposure was significantly higher in normal rats compared to AIA rats. C_max_, AUC_(0-t)_, and AUC_(0-∞)_ of SIN in normal rats were 2.01, 1.94, and 2.14 times higher than in AIA rats, respectively. In contrast, AIA rats exhibited a 2.24–fold increase in both CL/F and V/F compared to normal controls (*p* < 0.05), suggesting that systemic inflammation significantly enhanced the tissue distribution of SIN.

### 3.4. Absorption Studies Using Everted Gut Sac Model

**Figure 4:**
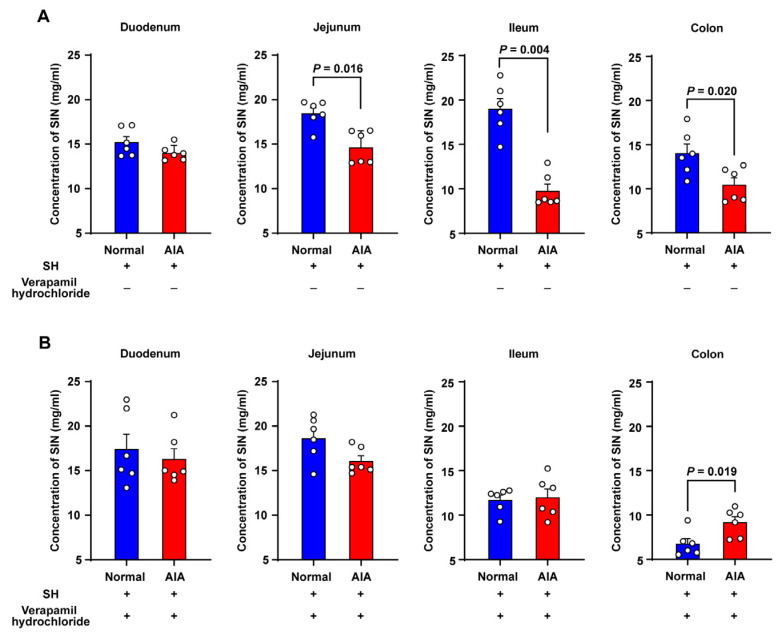
The absorptive characteristics of SH in the everted gut sac system of rats. (**A**) Krebs–Ringer buffer containing 60 mg/mL SH; (**B**) Krebs–Ringer buffer containing 60 mg/mL SH and 0.25 mg/mL verapamil hydrochloride. Data are presented as the means ± SEM (*n* = 6).

The everted gut sac model was employed to assess the impact of physiological and pathological conditions on SH absorption, quantifying its mucosal transport into the luminal contents of four intestinal segments: duodenum, jejunum, ileum, and colon. Quantitative analysis revealed significantly reduced SH transport in the jejunum, ileum, and colon of AIA rats versus normal controls, whereas duodenal transport remained comparable between groups (Figure 4A). To investigate the mechanistic basis for differential SH uptake, intestinal P–gp activity was examined by co–administering SH (60 mg/mL) with verapamil hydrochloride, a selective P–gp inhibitor. As depicted in Figure 4B, verapamil enhanced SH absorption across the small intestine. Surprisingly, markedly decreased SH absorption was observed in the ileum of normal rats.

### 3.5. Plasma Protein Binding

**Figure 5:**
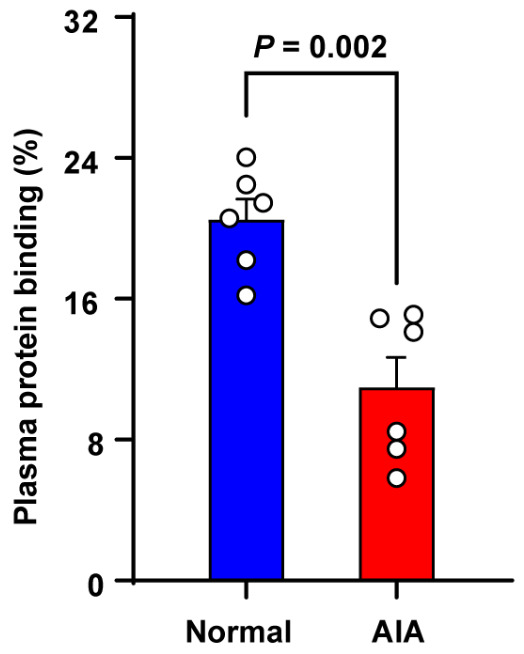
Plasma protein binding of SIN in SD female normal and AIA rats. Data are presented as the means ± SEM (*n* = 6).

The PPB of SIN was determined using the RED technique, and the results are presented in Figure 5. The PPB of SIN was markedly greater in normal rats compared to AIA rats.

## 4. Discussion

Epidemiological studies indicate that the prevalence of RA in men is approximately one–third that in women [29]. Therefore, female rats were chosen as the research subjects to enhance the clinical relevance of this study. Following an oral dose of SH, normal rats exhibited a higher risk of toxicity and mortality compared to AIA rats. The LD_50_ of SH was 1.5 times higher in AIA rats than in normal rats, highlighting that AIA rats displayed greater resistance to SH-induced toxicity. These findings highlight the varying susceptibility of rats to toxicants under different physiological and pathological states. Consequently, the different pathological conditions of patients should be considered when selecting and dosing therapeutic agents.

While the PK of SIN has been extensively explored, comparisons of its TK between normal and AIA rats have not been previously reported. Toxicity is often attributed to higher blood concentrations and reduced clearance of chemicals [24]. In this study, the TK characteristics of SIN were evaluated in both normal and AIA rats after administering an oral dose of 600 mg/kg SH. This dose was chosen based on the LD_50_ value of SH in normal rats (805.69 mg/kg), which is considerably higher than the clinically used dose of SH. The results revealed significant differences in most TK parameters of SIN, including C_max_, AUC, V/F, and CL/F, between the two groups. At most time points, SIN plasma concentrations were higher in normal rats than in AIA rats. Furthermore, normal rats exhibited significantly higher AUC and C_max_ values, indicating greater exposure to SIN in this group. In contrast, the V/F and CL/F values were 2.24-fold higher in AIA rats compared to normal rats, reflecting accelerated clearance of SIN in the pathological state.

To investigate the differences in SH absorption under physiological and pathological conditions in female rats, ex vivo everted gut sac models were employed. This method preserves the fundamental activities of intestinal cells and the mucus layers, making it a valuable tool for studying drug absorption in various intestinal segments [30,31]. The results demonstrated that SH absorption was evidently lower in pathological states compared to physiological states, indicating that pathological conditions impair intestinal drug absorption. P-gp, a transporter expressed in diverse tissues, plays a protective role by facilitating the excretion of endogenous and exogenous toxins. Furthermore, it has also been linked with changing the bioavailability of exogenous substances [32]. Previous studies have suggested that SIN might be a substrate of P–gp. For instance, Tsai and Wu [33] reported a marked increase in the bioavailability of SIN when co–administered with cyclosporin A, a P–gp inhibitor. P–gp is also known to regulate the hepatobiliary elimination of SIN. In pathological states, P–gp expression on the distal columnar epithelial surface of the small intestine in AIA rats was notably higher than in normal rats [34]. TNF–α, a pro–inflammatory cytokine integral to the pathogenesis of RA, can influence P–gp expression [35,36]. TNF–α triggers human intestinal mucosa to diminish P–gp mRNA expression while simultaneously upregulating P–gp expression in immune cells [37]. In this study, co–administration of verapamil hydrochloride notably increased SH absorption into the most intestinal sac contents. Under these conditions, no significant differences in SH uptake were noted between physiological and pathological states in the jejunum and ileum. These findings suggest that SH is poorly absorbed in pathological states primarily owing to the activity of efflux transporters such as P–gp, which modulate drug transport and reduce bioavailability. In addition, we observed a paradoxical reduction in SH absorption in the ileum of normal rats following verapamil treatment (Figure 4B). While verapamil is a well–characterized P–gp inhibitor, it also exhibits off–target inhibition of key intestinal uptake transporters, including OATP1A2, OCT1, and OCTN1 [38,39,40]. This paradoxical effect may be attributed to the competitive inhibition of these transporters which requires further investigation. Xenobiotics that bind to plasma proteins significantly influence their distribution, metabolism, and toxicity, as only the unbound fraction is capable of crossing cell membranes, interacting with biological targets, and undergoing cellular metabolic processes [41]. Further, PPB also plays essential physiological roles, including serving as a transporter and reservoir for exogenous substances [42]. An increase in PPB generally indicates a lower concentration of the free, pharmacologically active drug in plasma [43]. In this study, the RED technique was used to estimate the percentage of SIN bound to plasma proteins. Hepatic drug elimination is often limited by the extent of PPB [44], as only unbound drugs can undergo metabolism and excretion [45]. The PPB of SIN was 2 times greater in normal rats than in AIA rats (20% vs. 10%), which is consistent with TK findings showing a notably lower clearance rate in normal rats. However, the limited absolute change in free fraction (80% vs. 90%) implies a marginal increase in free SH concentration in AIA rats; therefore, future work is necessary to establish exposure-toxicity relationships. The efficacy and safety of drugs are closely linked to PPB, as it impacts distribution, half-life, clearance, and the potential for drug–drug interactions by enabling displacement of one drug by another [46]. Albumin, the most abundant plasma protein, plays a critical role in neutralizing toxins, transporting therapeutic drugs, and maintaining colloid osmotic pressure within blood vessels. SIN is likely to bind more strongly to albumin than to α–1–acid glycoprotein due to its acidic properties [20]. Furthermore, the capacity of drugs to bind endogenous albumin varies with physiological states, and serum albumin levels are notably reduced in patients with RA [47].

Collectively, the observed differences in TK characteristics, intestinal absorption of SH, and in vitro PPB between physiological and pathological states suggest that normal rats experience higher drug exposure compared to AIA rats. This discrepancy may be attributed to enhanced P–gp–mediated efflux of SH in the small intestine, which lowers absorption in AIA rats. Additionally, the relatively higher PPB in normal rats may contribute to the lower plasma clearance rate observed in these animals.

## 5. Conclusions

This study investigated the differences in toxicity following oral administration of SH in female rats under physiological and pathological conditions. Additionally, a TK study was implemented utilizing a validated UPLC–MS/MS method. The experimental results revealed that the LD_50_ of SH was 1.5 times greater in AIA rats than in normal rats, indicating greater resistance to SH toxicity in the pathological state. The TK analysis demonstrated that, compared with AIA rats, normal rats exhibited higher plasma concentrations of SIN and slower clearance rates. To elucidate the mechanisms underlying these differences, intestinal absorption and in vitro PPB studies were performed. The results showed that SH absorption was more efficient in normal rats, likely due to differences in the transport capabilities of efflux transporters such as P–gp. Additionally, the higher PPB observed in normal rats limited hepatic elimination processes, contributing to their increased vulnerability to SH toxicity. These findings suggest that normal rats are more susceptible to SH toxicity compared with AIA rats due to differences in drug transport and metabolism. Future studies are warranted to explore the effects of SIN metabolites on the expression and activity of intestinal P–gp to further clarify these mechanisms.

## Figures and Tables

**Table 1:** Linearity of SIN in different matrices harvested from SD female normal rats.

Tissue	Female Normal Rats	
	Standard Curve	Linearity
Plasma	Y = 0.00750X + 0.1500	r = 0.9916
Intestinal serosal fluid	Y = 0.00920X + 0.0314	r = 0.9974
Plasma protein binding	Y = 0.00879X + 0.0016	r = 0.9995

**Table 2:** Inter– and intra–day accuracy and precision, extraction recovery, and matrix effect of measurements of SIN in the intestinal serosal fluid of rats.

Matrix	Concentration (ng/mL)	Intra–Day (*n* = 6)		Inter–Day (*n* = 18)		Extraction Recovery (%) (Mean ± SD, *n* = 6)	Matrix Effect (%) (Mean ± SD, *n* = 6)
		Accuracy (%)	Precision RSD (%)	Accuracy (%)	Precision RSD (%)		
Intestinal serosal fluid	200.00	99.50	4.63	106.03	6.10	97.03 ± 2.39	98.15 ± 2.80
	350.00	99.50	1.68	105.40	4.43	104.05 ± 2.49	104.26 ± 8.76
	700.00	102.62	2.11	100.20	2.95	98.83 ± 3.33	101.01 ± 1.43

**Table 3:** Stability of SIN in differently stored intestinal serosal fluid samples of rats (*n* = 6).

Matrix	Condition	Nominal Concentration (ng/mL)	Measured Concentration (mean ± SD, ng/mL)	RSD Precision (%)
Intestinal serosal fluid	Room temperature stability	200.00	222.17 ± 1.94	0.87
		350.00	356.17 ± 6.62	1.86
		700.00	649.50 ± 14.61	2.25
	Freeze–thaw stability	200.00	204.50 ± 6.47	3.17
		350.00	371.17 ± 4.58	1.23
		700.00	702.83 ± 10.48	1.49
	Long term freezing stability	200.00	229.67 ± 4.76	2.07
		350.00	386.33 ± 7.00	1.81
		700.00	689.50 ± 11.50	1.67

**Table 4:** Comparison of the toxicokinetic parameters of oral administration of 600 mg/kg SH in SD female normal and AIA rats (Mean ± SD, *n* = 6).

TK Parameter	Female Normal Rats	Female AIA Rats	*p*-Value
T_max_ (min)	73.3 ± 58.5	86.7 ± 97.7	0.746
C_max_ (ng/mL)	54,500.0 ± 5950.8	27,105.0 ± 6689.2	0.000
T_1/2_ (min)	476.9 ± 259.1	425.6 ± 129.4	0.070
AUC_(0-t)_ (ng·min/mL)	21,157,950.0 ± 5,011,214.1	10,927,737.5 ± 4,038,606.3	0.003
AUC_(0-∞)_ (ng·min/mL)	34,574,304.2 ± 8,516,605.5	16,168,486.3 ± 5,832,812.7	0.001
CL/F (mL/min/kg)	18.4 ± 5.4	41.3 ± 14.8	0.005
V/F (mL/kg)	11,537.8 ± 4686.3	25,788.8 ± 13,299.8	0.047
ka (h^−1^)	1.32 ± 1.44	1.38 ± 0.92	0.749
ke (h^−1^)	0.11 ± 0.06	0.10 ± 0.03	0.798

C_max_ maximum concentration, T_max_ time to reach C_max_, T_1/2_ elimination half-life, AUC area under the curve, CL/F apparent clearance, V/F volume of distribution, ka: absorption rate constant, ke: elimination rate constant.

## Data Availability

Data are contained within the article.
